# Household vacuum cleaners vs. the high-volume surface sampler for collection of carpet dust samples in epidemiologic studies of children

**DOI:** 10.1186/1476-069X-7-6

**Published:** 2008-02-21

**Authors:** Joanne S Colt, Robert B Gunier, Catherine Metayer, Marcia G Nishioka, Erin M Bell, Peggy Reynolds, Patricia A Buffler, Mary H Ward

**Affiliations:** 1 Division of Cancer Epidemiology and Genetics, National Cancer Institute, National Institutes of Health, Department of Health and Human Services, 6120 Executive Blvd., MSC 7240, Bethesda, MD, 20892-7240, USA; 2 Northern California Cancer Center, 2001 Center Street, Suite 700, Berkeley, CA, 94704, USA; 3 School of Public Health, University of California, 50 University Hall, MC 7360, Berkeley, CA, 94720-7360, USA; 4 Battelle Memorial Institute, 505 King Avenue, Columbus, OH, 43201, USA; 5 Department of Epidemiology, University at Albany, School of Public Health, One University Place, Room 130, Rensselaer, NY, 12144, USA

## Abstract

**Background:**

Levels of pesticides and other compounds in carpet dust can be useful indicators of exposure in epidemiologic studies, particularly for young children who are in frequent contact with carpets. The high-volume surface sampler (HVS3) is often used to collect dust samples in the room in which the child had spent the most time. This method can be expensive and cumbersome, and it has been suggested that an easier method would be to remove dust that had already been collected with the household vacuum cleaner. However, the household vacuum integrates exposures over multiple rooms, some of which are not relevant to the child's exposure, and differences in vacuuming equipment and practices could affect the chemical concentration data. Here, we compare levels of pesticides and other compounds in dust from household vacuums to that collected using the HVS3.

**Methods:**

Both methods were used in 45 homes in California. HVS3 samples were collected in one room, while the household vacuum had typically been used throughout the home. The samples were analyzed for 64 organic compounds, including pesticides, polycyclic aromatic hydrocarbons, and polychlorinated biphenyls (PCBs), using GC/MS in multiple ion monitoring mode; and for nine metals using conventional microwave-assisted acid digestion combined with ICP/MS.

**Results:**

The methods agreed in detecting the presence of the compounds 77% to 100% of the time (median 95%). For compounds with less than 100% agreement, neither method was consistently more sensitive than the other. Median concentrations were similar for most analytes, and Spearman correlation coefficients were 0.60 or higher except for allethrin (0.15) and malathion (0.24), which were detected infrequently, and benzo(k)fluoranthene (0.55), benzo(a)pyrene (0.55), PCB 105 (0.54), PCB 118 (0.54), and PCB 138 (0.58). Assuming that the HVS3 method is the "gold standard," the extent to which the household vacuum cleaner method yields relative risk estimates closer to unity by increasing random measurement error varies by compound and depends on the method used to calculate relative risk.

**Conclusion:**

The household vacuum cleaner method appears to be a reasonable alternative to the HVS3 for detecting, ranking, and quantifying the concentrations of pesticides and other compounds in carpet dust.

## Background

Pesticides may enter the home from indoor use, track-in or drift from outdoors, or take-home contamination from occupational use [1-3]. Pesticides may persist for long periods of time inside the home, where they are protected from degradation by sunlight, rain, temperature extremes, and most microbial action [4]. Carpets are repositories for pesticides [5-7]; the fibers and underlying foam pad appear to act as long-term reservoirs that continuously transfer pesticides to carpet dust [8,9]. Several large epidemiology and exposure studies have collected carpet dust samples for analysis for pesticides and other compounds, including the Long Island Breast Cancer Study [10], the National Cancer Institute-Surveillance, Epidemiology, and End Results Case-Control Study of non-Hodgkin lymphoma [11], and the Center for the Health Assessment of Mothers and Children of Salinas cohort study [12].

Young children, who spend a large proportion of time on the floor and who frequently put hands and objects in their mouths [7], are at particularly high risk of exposure to pesticides in carpet dust. The possible health effects of such exposures have been investigated in epidemiologic studies using a variety of exposure assessment techniques, including use of a specially-designed vacuum cleaner called the high-volume surface sampler (HVS3, Cascade Sampling Systems, Bend, Oregon) [13,14] to collect carpet dust samples in the child's home. The HVS3 is designed to collect particles >5 microns in diameter and to achieve constant dust removal efficiency across different types of carpet, allowing an estimation of chemical loading (mass of chemical per unit surface area of carpet). For some metals, there is some evidence that loading is a better indication of dose than is concentration; in studies of children's exposure to lead, the surface loading of lead on carpet was more strongly correlated with blood lead levels than was the concentration of lead in the dust [15,16].

The HVS3 and associated supplies are expensive; the equipment can be difficult to use and clean, and the sample collection process is time consuming and labor intensive. An alternative sampling method is to remove the dust that has already been collected using the household vacuum cleaner and to sample a portion of the dust in the laboratory. Although this method does not allow for the estimation of chemical loading, it provides a simplified method for collecting dust samples for the purpose of measuring pesticide and other chemical concentrations.

We previously conducted a small study to determine whether the quality of the chemical concentration data in dust samples collected from used vacuum cleaner bags would be compromised by variations in peoples' vacuuming equipment and practices and by the repeated passage of air through the dust bag during multiple uses of the vacuum [17]. In each of 15 homes, we collected carpet dust samples using the HVS3 and also removed the used bag from the household vacuum. The HVS3 sample was collected in all of the rooms in which the household vacuum had been used, adjusting the HVS3 square footage in each room to reflect the frequency with which that room had been vacuumed by the homeowner; we did this to ensure that the HVS3 and used bag samples were composed of similar proportions of dust from each room. The samples were analyzed for 26 pesticides, 10 polycyclic aromatic hydrocarbons (PAHs), and six polychlorinated biphenyl (PCB) congeners using gas chromatography/mass spectrometry (GC/MS) in selected ion monitoring mode. We found no clear difference in the pesticide, PAH, or PCB concentration data between the two dust collection methods.

While the above study was designed to compare concentrations of pesticides and other chemicals in dust samples collected by two different types of vacuums, it did not reflect how the HVS3 is typically used in epidemiologic studies. In practice, the HVS3 is rarely used in more than one room. Not only is it time consuming to sample more than one room, but researchers are typically interested in focusing on the room that accounts for the highest proportion of the child's exposure. Sampling dust that has already been collected using the household vacuum cleaner precludes the ability to single out that room, and researchers who are choosing between the two methods must consider the imprecision introduced by using a "whole-house" sample instead. The current study addresses this issue by comparing dust removed from household vacuum cleaners with HVS3 samples collected in a single room. It also addresses two limitations of the previous study by increasing the sample size and adding agricultural pesticides and metals to the list of measured compounds.

## Methods

### Study households

We selected households from two ongoing studies. One is a case-control study of childhood leukemia in Northern California (the Northern California Childhood Leukemia Study [NCCLS]), which includes 17 counties in the San Francisco Bay area and 18 counties in the Central Valley, as described previously [18]. Beginning in 2001, interviewed cases and controls under 8 years old who were living at the home they occupied at the time of diagnosis (or a similar reference date for controls) were eligible for a second home visit in which we collected detailed information on home and garden pesticide use, inventoried pesticides in storage, and collected carpet dust samples using the HVS3 and from household vacuum cleaners. The second study was conducted in Fresno County, CA. It was designed to examine the validity of agricultural metrics [19] for estimating exposure to pesticides in residences located near crops. Carpet dust samples were collected using both methods, and interviewers obtained information on home and garden pesticide use and occupational pesticide exposure. The study protocols were approved by the Institutional Review Boards of all collaborating institutions, and written informed consents were obtained for all participating subjects.

For the current study, we had sufficient resources to analyze samples from 45 of the 148 households in both studies for which carpet dust samples had already been collected using both methods. We ruled out households if there was insufficient HVS3 dust to support the extractions and analyses needed for both this substudy and the two main studies (n = 74), or if either the HVS3 or vacuum cleaner sample from a given home had been lost due to logistical problems (n = 24). From the 54 remaining households, we selected 19 located in agricultural areas (12 from the Fresno study and 7 from the NCCLS), defined as having at least 40 acres of crops within 500 meters of the household. To determine proximity to agricultural activities, we recorded residence locations using a hand-held Garmin E-trex Legend global positioning system (Garmin International Inc., Olathe, KS), and then mapped the residences on crop maps created by the California Department of Water Resources or the National Land Cover Dataset, if the former was unavailable. We used these maps to estimate the acres of cropland or pasture within 500 meters of the household. The remaining 26 households were from non-agricultural areas in the NCCLS. The nine households that were not included in the study were of less interest for a variety of reasons, such as having little or no reported pesticide use.

### Dust collection

In the NCCLS, we asked parents to identify the room (other than the kitchen or the child's bedroom) in which the child had spent the most time, while awake, during the year before diagnosis or reference date. If that room had a carpet or area rug measuring at least 9 square feet that was present before the reference date, and if there was adequate space in the room to manipulate the HVS3, we took the HVS3 sample there. Otherwise, another room was selected instead. For most subjects, the sample room was the living room or family room. For the Fresno study, the room was selected from those located on the side of the home facing agricultural crops. If the first room contained a carpet or area rug that was large enough, an HVS3 samples was taken. Otherwise, another room on the same side of the home was selected instead.

To collect the HVS3 dust sample, the nozzle was leveled and the flow rate and suction were adjusted for each type of carpet vacuumed. An approximately 4-foot by 6-foot area was selected in an open area of the room, preferably at a location where the child had played. If there was more than one rug in the room, the rug that had been in the room the longest was selected. The interviewer marked off the area with measuring tapes and the surface was vacuumed in 3-inch strips, making four passes back and forth on each strip. Interviewers were instructed to continue vacuuming until the level of fine dust in the Teflon catch bottle reached a pre-marked level of 10 mL. Additional areas in the room were sampled if necessary to collect the desired amount of dust. The HVS3 sampling train was cleaned with isopropyl alcohol and dried between uses at each different home.

In addition, we removed the used bag from the household vacuum cleaner and placed it in a sealable polyethylene bag. If the household vacuum did not have a bag (22%), the loose dust was emptied directly into a sealable polyethylene bag. The polyethylene bag and the sealed Teflon collection bottle containing the HVS3 dust sample were labeled with the collection date and subject identification number and shipped via overnight mail in a styrofoam container with ice packs to Southwest Research Institute (San Antonio, TX), where they were placed in freezers (-12°C). They were subsequently shipped to Battelle Memorial Institute (Columbus, OH) and stored in freezers (-20°C) prior to analysis.

### Chemical analysis

The dust samples were sieved using a 100 mesh stainless steel sieve to remove the coarse (>150 μm) fraction of dust. To cover the broad suite of organic analytes of interest, we used three different extraction methods, each combined with GC/MS in the multiple ion detection mode, for detection and quantification. Sample batches consisted of 15 field samples plus three quality control (QC) samples: a lab duplicate, a solvent method blank, and a duplicate spiked sample (spiked with either 50 ng or 250 ng) or solvent spiked sample (100 ng/analyte). For analysis of metals, conventional microwave-assisted acid digestion was combined with ICP/MS. Sample batches consisted of 35 to 37 field samples, one SRM (NIST 2583), a method blank, a duplicate, and a duplicate spiked sample (spiked to give 2.5 μg/L in the extract for all analytes except zinc, which was spiked to give 25 μg/L); in addition, three digested samples were analyzed in duplicate and then spiked and reanalyzed.

Resource constraints precluded us from performing all analyses on every sample, so we selected 15 sample pairs for both neutral and acid extractions, 25 for neutral extractions only, and 5 for acid extractions only (for a total of 40 sample pairs extracted for neutrals and 20 for acids). We chose a larger sample size for the neutral extractions because there were 10-fold more neutral-extractable analytes (n = 64) than acid-extractable analytes (n = 6). We extracted metals from all samples with sufficient dust remaining after the neutral and/or acid extractions (n = 19).

For the hexane:acetone (H:A) extraction method, a 0.5 g aliquot of fine dust was spiked with 250 ng each of 14 surrogate recovery standards (SRSs), most of these being C13 labelled analogs of one of the analytes. The SRSs represent all major compound classes of the analytes and are added as a sample-by-sample check on method performance for the compound classes being analyzed. The dust was extracted with 12 mL of 1:1 H:A in an ultrasonic bath (Branson 5210) for 10 minutes. After centrifugation (Forma Scientific), a 10 mL aliquot was removed and concentrated to 1 mL. The extract was solvent exchanged into hexane and applied to a silica SPE cartridge (6 mL, 1000 mg loading; Baker) that had been conditioned in sequence with 20% acetone in ethyl acetate, dichloromethane, 15% diethyl ether in hexane, and hexane. The analytes were then eluted with these solvents in reverse order. The first three fractions were collected as one and concentrated to 1 mL; the final eluent was collected separately and concentrated to 1 mL. Two internal standards (IS) were added: p,p = -dibromophenyl (for pesticide and PCB analyses) and d_12_-benzo(e)pyrene (for PAH analyses).

The dichloromethane (DCM) method was similar to the H:A method, except the SRS mixture had two components rather than 14, the extraction was performed with DCM rather than H:A, the concentrated extract was solvent exchanged into ethyl acetate, there was no SPE cleanup step, and there was only one IS (p,p = -dibromophenyl).

For the herbicide acid (Acid) method, a 0.5 g aliquot of dust was weighed into a 60 mL centrifuge tube and 250 ng of one SRS was added. A 25 mL aliquot of the extraction solvent, 70:30 acetonitrile:phosphate buffer (0.1 M sodium acid phosphate) at pH = 3, was added to the dust. The dust was sonicated and centrifuged. A 20 mL aliquot of the extract was transferred to a separatory funnel containing deionized water, and the pH was adjusted to 1 with concentrated HCl. The aqueous layer was applied to a C18 SPE cartridge (6 mL, 500 mg; Baker) that had been conditioned just prior to use with methanol, deionized water, and 1:10 acetonitrile:0.025 M phosphoric acid. The extract was applied to the SPE cartridge and the cartridge was dried for two hours. The cartridge was eluted with 1:1 hexane:diethyl ether. The eluent was concentrated to near dryness under a stream of dry N_2_, resuspended in 5% methanol in methyl-t-butyl ether, and then methylated using ethereal diazomethane generated *in-situ *from Diazald, carbitol, and 37% aqueous potassium hydroxide. After the solutions were allowed to stand for 30 minutes, they were purged of excess diazomethane and the IS (p,p = -dibromobiphenyl) was added.

The H:A sample extracts were analyzed using an RTx-5 MS column (30 M, 0.25 mm id, 0.25 μm film) with a GC oven temperature programmed from 130–220°C @2°/min and then 220–330°C @10°/min. The DCM and Acid sample extracts were analyzed using a DB-1701 column (30 M, 0.25 mm id, 0.15 μm film) with the GC oven temperature programmed 130–220°C @2°C/min and then 220–280°C @10°/min for the DCM method, and programmed 140–280°C @20°/min for the Acid method. Typically two ions were monitored for each analyte, although for selected compounds (e.g., malathion) three ions were monitored for identification. An 8-point calibration curve, spanning the range of 2–750 ng/mL for analytes and 10–300 ng/mL for SRSs, plus an instrument blank, was analyzed concurrently with each sample set for each analytical method. Linear regression analysis was used to establish the calibration curve for each analyte.

For analysis of metals, 0.2 g of dust was digested in 10 mL of ultra-pure nitric acid in a Teflon microwave digestion vessel. Once capped, the vessels were heated slowly to 150°C and allowed to digest for three hours. After cooling, the digestate was transferred to a 50 mL conical tube and diluted to 50 mL with deionized water. Further dilutions were an additional 10X and 100X from the 50 ml volume. Solutions were analyzed in reverse order of dilution (e.g., 100X first) to obtain data without matrix effects and matched to the calibration range. The ICP/MS was calibrated daily using an 8 to 11 point calibration curve ranging in concentrations from 0.1 to 2,500 μg/mL. Internal standards, added in-line to samples and standards, were used for quantification and to correct for variations in instrument response. Quantification was performed using a linear regression analysis of the calibration curve data.

The analytes for which each extraction method was used are given in Table 1. Several additional analytes were tested for but not detected in these samples: beta-endosulfan, heptachlor, azinphos-methyl, dimethoate, methidathion, metolachlor, ethafluralin, cyanazine, butylate, and pebulate, with detection limits 10 to 100 ng/g in the H:A method; and prometryn, phorate, tribufos, and bromoxynil octanoate, with detection limits 10 to 50 ng/g in the DCM method. These analytes are not listed on Table 1 and are not considered further in this paper. Spiking of four randomly selected dust samples with 250 ng (500 ng/g) of each organic analyte showed that among the 64 organic analytes detected in at least one household, mean recoveries generally ranged from 81% to 125%. Analytes with lower recovery means were dicamba (33 ± 20%), chlorothalonil (48 ± 55%), acephate (69 ± 21%), mecoprop (MCPP) (69 ± 35%), and piperonyl butoxide (73 ± 11%); and analytes with higher recovery means were allethrin 1 (127 ± 21%), allethrin 2 (136 ± 31%), carbaryl (130 ± 36%), cyfluthrin 4 (131 ± 27%), malathion (134 ± 26%), iprodione (139 ± 27%), phosmet (140 ± 21%), cypermethrin 2 (141 ± 19%), cypermethrin 4 (141 ± 24%), methoxychlor (145 ± 42%), and dichlorodiphenyltrichloroethane (p,p'-DDT) (163 ± 26%). Three additional analytes had mean recovery standard deviations exceeding 40% (propargite: 111 ± 51%, deltamethrin: 125 ± 52%, and dicofol: 103 ± 86%). The SRS recoveries averaged from 85 to127% in these QC samples, as well as in field samples, with the exception of ^13^C_12_-p,p'-DDT (139 ± 13%), F_4_-terephthalonitrile (53 ± 14%), and d_4_-cotinine (65 ± 26%). Laboratory spikes with metals at 60 μg/g showed mean recoveries ranging from 86 to 100%, with mean standard deviations of 3 to 7%. We did not adjust reported analyte levels in dust for spike recoveries or SRS recoveries in this analysis. In addition to the spiked dust samples, up to seven samples were analyzed in duplicate. The average relative percent difference for duplicates was typically 10 to 30% for organics and 1 to 10% for the metals, indicating very good agreement between pairs of samples.

**Table 1:** Comparison of the HVS3 and Household Vacuum Sampling Methods in Detecting and Ranking Concentrations of Chemicals

Class	Analyte	Method^a^	Method Detection Limit^b^	N^c^	No. of Homes in Which Detected		Percent Agreement in Detection	Spearman Rank Correlation Coefficient	Pearson Correlation Coefficient	Observed Odds Ratio^e^
									
					HVS3	HH Vac^d^				
Organochlorine Insecticides	γ-Chlordane	H:A	2	40	39	36	93%	0.83	0.86	1.7–1.8
	α-Chlordane	H:A	2	40	38	35	88%	0.78	0.79	1.6–1.7
	p,p'-DDE	H:A	2	40	35	34	83%	0.81	0.72	1.4–1.6
	p,p'-DDT	H:A	10	39	21	24	77%	0.66	0.67	1.3–1.6
	Dieldrin	H:A	50	40	2	4	95%	0.69	0.64	1.3–1.6
	Dicofol	H:A	50	40	1	1	100%	--	--	--
	Lindane	H:A	10	40	1	0	98%	--	--	--
	Methoxychlor	H:A	10	40	14	14	90%	0.84	0.88	1.8–1.9
	Pentachlorophenol	Acid	5	20	20	20	100%	0.63	0.82	1.6–1.7
Organophosphate insecticides	Acephate	H:A	100	40	1	1	100%	--	--	--
	Chlorpyrifos	H:A	5	40	40	38	95%	0.67	0.78	1.6–1.7
	Diazinon	H:A	2	40	35	37	85%	0.76	0.77	1.5–1.7
	Phosmet	H:A	25	40	16	14	90%	0.90	0.95	1.8–1.9
	Malathion	H:A	10	40	3	3	90%	0.24	0.04	1.0–1.1
	Methyl parathion	H:A	20	40	3	2	98%	0.83	0.82	1.6–1.7
Carbamate insecticides	Carbaryl	H:A	2	40	35	37	95%	0.71	0.67	1.3–1.6
	Propoxur	H:A	5	39	33	33	85%	0.60	0.57	1.2–1.5
Pyrethroid insecticides	Allethrin 1^f^	H:A	20	40	6	3	83%	0.15	0.16	1.0–1.1
	Allethrin 2	H:A	20	40	6	3	83%	0.15	0.14	1.0–1.1
	Cypermethrin 1	H:A	20	40	17	16	83%	0.81	0.91	1.8–1.9
	Cypermethrin 2	H:A	20	40	18	18	85%	0.83	0.91	1.8–1.9
	Cypermethrin 3	H:A	20	40	18	18	85%	0.77	0.87	1.7–1.8
	Cypermethrin 4	H:A	20	40	17	16	83%	0.77	0.89	1.8–1.9
	Cyfluthrin 1	H:A	20	38	7	6	87%	0.61	0.70	1.4–1.6
	Cyfluthrin 2	H:A	20	40	8	6	90%	0.67	0.66	1.3–1.6
	Cyfluthrin 3	H:A	20	40	8	7	88%	0.69	0.77	1.5–1.7
	Cyfluthrin 4	H:A	20	40	8	6	90%	0.71	0.72	1.4–1.6
	Deltamethrin	H:A	50	40	2	4	95%	0.69	0.69	1.4–1.6
	cis-Permethrin	H:A	2	40	40	40	100%	0.87	0.87	1.7–1.8
	trans-Permethrin	H:A	2	40	40	40	100%	0.83	0.83	1.7–1.8
	Tetramethrin 1	H:A	2	40	4	6	95%	0.80	0.81	1.6–1.7
	Tetramethrin 2	H:A	2	40	5	8	88%	0.62	0.70	1.4–1.6
Phenoxy herbicides	2,4-D	Acid	5	20	17	17	100%	0.74	0.89	1.8–1.9
	MCPA	Acid	5	20	2	2	100%	1.00	1.00	2.00
	MCPP	Acid	5	20	2	3	95%	0.84	0.89	1.8–1.9
Amide herbicides	Alachlor	H:A	10	40	1	1	100%	--	--	--
	Iprodione	H:A	20	39	13	16	87%	0.78	0.80	1.6–1.7
Dinitroaniline herbicides	Pendimethalin	H:A	10	40	1	1	100%	--	--	--
	Trifluralin	H:A	2	40	18	18	85%	0.87	0.95	1.8–1.9
Other herbicides	Bromoxynil	Acid	2	20	5	2	85%	0.68	0.75	1.5–1.7
	Dacthal	H:A	1	40	10	10	90%	0.68	0.60	1.3–1.5
	Dicamba	Acid	5	20	1	1	100%	--	--	--
	Simazine	H:A	2	40	25	26	78%	0.75	0.76	1.5–1.7
Other pesticides	Chlorothalonil	H:A	10	39	4	4	95%	0.68	0.57	1.2–1.5
	Methoprene	H:A	100	40	0	1	98%	B	--	--
	o-Phenylphenol	H:A	10	40	38	40	95%	0.64	0.47	1.2–1.4
	Propargite	DCM	100	40	2	2	100%	1.00	0.99	>1.9
PAHs	Benzo(a)anthracene	H:A	2	40	40	40	100%	0.69	0.89	1.8–1.9
	Benzo(b)fluoranthene	H:A	2	40	40	40	100%	0.68	0.85	1.7–1.8
	Benzo(k)fluoranthene	H:A	2	40	40	40	100%	0.55	0.68	1.4–1.6
	Benzo(a)pyrene	H:A	2	40	40	40	100%	0.55	0.71	1.4–1.6
	Chrysene	H:A	2	40	40	40	100%	0.78	0.84	1.7–1.8
	Coronene	H:A	4	40	39	39	95%	0.81	0.78	1.6–1.7
	Dibenzo(ae)pyrene	H:A	4	38	37	33	89%	0.70	0.77	1.5–1.7
	Dibenz(ah)anthracene	H:A	2	40	34	35	93%	0.67	0.75	1.5–1.7
	Indeno(123cd)pyrene	H:A	2	40	40	40	100%	0.77	0.89	1.8–1.9
PCBs	PCB 105	H:A	1	40	12	11	83%	0.54	0.46	1.2–1.4
	PCB 118	H:A	1	40	10	11	83%	0.54	0.49	1.2–1.4
	PCB 138	H:A	1	40	17	16	78%	0.58	0.49	1.2–1.4
	PCB 153	H:A	1	40	23	21	90%	0.83	0.76	1.5–1.7
	PCB 170	H:A	2	40	7	7	90%	0.63	0.56	1.2–1.5
	PCB 180	H:A	2	40	19	20	83%	0.82	0.83	1.7–1.8
Metals	Arsenic	Metals	0.25	19	19	19	100%	0.68	0.53	1.2–1.5
	Cadmium	Metals	0.25	19	19	19	100%	0.73	0.69	1.4–1.6
	Chromium	Metals	2.5	19	19	19	100%	0.79	0.85	1.7–1.8
	Copper	Metals	0.25	19	19	19	100%	0.76	0.79	1.6–1.7
	Lead	Metals	0.25	19	19	19	100%	0.85	0.71	1.4–1.6
	Nickel	Metals	0.25	19	19	19	100%	0.64	0.73	1.5–1.7
	Tin	Metals	2.5	19	14	13	84%	0.70	0.82	1.6–1.7
	Tungsten	Metals	0.25	19	0	1	95%	NA	--	--
	Zinc	Metals	25	19	19	19	100%	0.85	0.81	1.6–1.7
Other compounds	Cotinine	DCM	10	40	10	12	90%	0.80	0.85	1.7–1.8
	Piperonyl butoxide	DCM	4	40	37	38	93%	0.80	0.70	1.4–1.6

^a^H:A = hexane:acetone method; DCM = dichloromethane method.

^b^ug/g for metals; ng/g for all other analytes.

^c^N = Number of samples included in all statistical analyses for that analyte. N excludes samples in which the analyte level could not be determined because of the presence of interfering compounds.

^d^HH Vac = household vacuum cleaner method.

^e^Assuming a true odds ratio of 2.0.

^f^First isomer to elute chromatographically.

### Statistical Analysis

Comparison of the two dust collection methods was performed on a compound-by-compound basis. If the compound was not detected by GC/MS, we set the concentration equal to half of the method detection limit. Infrequently, compounds were detected at levels below the method detection limit; we considered these to be non-detects and adjusted their concentrations to half of the method detection limit.

We calculated the percent agreement in detection of each compound between the two types of dust samples. We performed a paired t-test on the log transformed values for each compound, excluding pairs where the compound was not detected in either sample. We also calculated a Spearman rank correlation coefficient for each compound that was detected in more than two sample pairs.

Finally, using the analyte concentration in the HVS3 sample as the Agold standard,@ we evaluated what effect the random measurement error introduced by the household vacuum sampling method would have on the estimate of relative risk in a case-control study. We used a table from de Klerk et al. [20], in which the authors provide values for observed relative risk for various combinations of true relative risk, Pearson correlation coefficient, and anticipated method of calculating relative risk. Four methods for calculating relative risk are shown: logistic method (odds ratio per unit increment in a logistic regression model), fixed value method (risk of some fixed value in the control distribution relative to some lower value expressed in terms of the variation of the distribution itself, such as the risk of one standard deviation above the mean relative to the mean value), extreme quantile method (risk of the top quantile group of the exposure distribution for controls relative to the bottom quantile group), and median method (risk of the top half of the exposure distribution among controls relative to the bottom half).

In applying this method, we calculated the Pearson correlation coefficient for each compound using log transformed values. Among the correlation coefficients presented by de Klerk et al. (intervals of 0.05), we chose the one that was closest to the actual coefficient. We present the observed relative risk as a range across the four methods for calculating relative risk. For most values of the correlation coefficient (i.e,. ≤ 0.90), the lower end of the range corresponds to the logistic method and upper end to the fixed value method.

## Results

Method detection limits ranged from 1–100 ng/g for pesticides, 1–2 ng/g for PCBs, 2–4 ng/g for PAHs, and 0.25–25 μg/g for metals (Table 1). The two methods of dust collection agreed in detecting the presence of the target compounds between 77% and 100% of the time (median 95%). For compounds with less than 100% agreement, neither method was consistently more sensitive than the other in detecting the presence of a compound. Paired t-tests showed no significant differences in the concentrations between the methods for any of the analytes except o-phenyphenol (geometric mean 1.5 times higher in vacuum bag samples, p = 0.02) and benzo(a)pyrene (geometric mean 1.2 times higher in vacuum bag samples, p = 0.01) (not shown).

The Spearman correlation coefficients were moderate to high (0.60 or higher) for most compounds. Spearman correlations were lower for allethrin (0.15) and malathion (0.24), which were detected infrequently, as well as for benzo(k)fluoranthene (0.55), benzo(a)pyrene (0.55), PCB 105 (0.54), PCB 118 (0.54), and PCB 138 (0.58). The Pearson correlation coefficients were similar to the Spearman correlation coefficients for most compounds (Table 1).

Using the HVS3 sampling method as the "gold standard" and assuming that the true relative risk for the disease under study is 2.0, the relative risks that might be observed if the dust were collected from household vacuum cleaners are shown in Table 1. If relative risk is calculated using the fixed value method (the upper end of the range in Table 1), the relative risk is above 1.5 for most compounds in this study; those with observed relative risks below 1.5 are malathion (1.1), allethrin (1.1), o-phenylphenol (1.4), and PCBs 105, 118, and 138 (1.4). Several others would be added to that list if risk is calculated using the logistic method.

We recalculated the statistics after excluding the10 homes whose household vacuum did not have a removable bag (data not shown). Compared to the results in Table 1, the difference in percent agreement was less than 10% for all compounds except PCB 105 (increased from 83% to 93%) and PCB 170 (increased from 90% to 100%). The Spearman and Pearson correlation coefficients increased for 43 analytes and decreased for 15 (for the remainder, the coefficients either remained the same or could not be calculated). Spearman correlations for methyl parathion, propoxur, cyfluthrin, deltamethrin, tetramethrin, dibenzo(a,e)pyrene, PCB 105, PCB 170, arsenic, cadmium, chromium, and nickel increased by 10% or more; decreases of 10% or more were observed for dieldrin, copper, and tin. Increases in the Pearson correlation coefficient result in increased observed relative risks for many analytes. For the six analytes with observed relative risks below 1.5 using the fixed value method, deleting the loose dust samples from the data set had no effect for malathion or o-phenylphenol, but resulted in higher ranges of observed relative risks for allethrin (1.1–1.3) and the three PCB congeners (1.8–1.9 for PCB 105, 1.4–1.6 for PCB 118 and PCB 138).

## Discussion

As in the earlier study, the two dust collection methods performed similarly in detecting compounds in carpet dust, and neither was consistently more sensitive than the other. A paired t-test showed no significant differences in concentrations for most analytes. The Spearman rank correlation coefficients were moderate to high for most compounds, indicating that homes would be ranked in a similar order using either method. The two methods compared well for agricultural pesticides and metals, which were not included in the earlier study.

For most of the analytes that were included in both studies, the Spearman correlations were lower in the current study. This likely reflects the added imprecision associated with comparing a single-room HVS3 sample with a "whole-house" vacuum cleaner sample, both of which are consistent with typical field practice. The agreement measures in the current study therefore reflect differences in both the equipment used and the dust composition. In addition, unlike the previous study, the current study includes loose dust samples from household vacuums without removable bags, likely contributing to the lower observed correlations.

In the current study, Spearman correlations were quite low for the pesticides malathion and allethrin. Malathion was detected in three HVS3 samples and three household vacuum cleaner samples, but in only one household was it was detected in both samples. Allethrin was detected in six HVS3 samples and three household vacuum cleaner samples, again with only one household with detections in both. In the earlier study, allethrin was not on the target analyte list and malathion was not detected in any of the samples.

Ortho-phenylphenol, a commonly used fungicide and disinfectant, had poor correlations in both studies. The Spearman correlation coefficient was higher here (0.64 compared to 0.46), but the low Pearson correlation coefficient (0.47) indicates that the relative risk estimate would be significantly affected. We observed a much stronger correlation for the herbicide 2,4-D in the current study than in the earlier one (Spearman correlations 0.74 and 0.37, respectively). There were more detections in the current study (17 of 20 samples compared to 8 to 9 of 15 household vacuum and HVS3 samples, respectively) and a much wider concentration range among the detected values (172-fold vs. 5-fold).

The Spearman correlations for the PAHs were considerably lower in the current study (0.55 to 0.81) than in the previous one (0.85 to 0.94). They were lowest for benzo(k)fluoranthene and benzo(a)pyrene (0.55). However, the Pearson correlations for these two compounds were 0.68 and 0.71, respectively, and therefore the estimated effect on the relative risk estimate was only moderate. For these and the other PAHs, the two methods agreed quite well when limited to samples with relatively high PAH levels.

Correlations for three of the PCB congeners (105, 118, and 138) were relatively low (less than 0.60). Removing from the analysis the households with loose dust samples resulted in higher Spearman correlations. In the earlier study, PCB 118 was not among the analytes measured, and PCBs 105 and 138 were not detected in enough samples to be included in the correlation analysis. Results for the other three congeners were consistent across the two studies.

In the earlier study, we noted that for many analytes, concentrations in the HVS3 samples exceeded those in the household vacuum samples at the upper end of the concentration ranges. This was not observed in the current study.

If the HVS3 concentration is viewed as the "gold standard," the nondifferential misclassification of exposure introduced by use of the household vacuum cleaner method yields relative risk estimates that are closer to unity. The extent to which this is expected to occur varies by compound and depends on the method used to calculate relative risk. For a small number of compounds (malathion, allethrin, o-phenylphenol, and PCBs 105, 118, and 138), our analysis shows that a true positive association would be observed to be much closer to unity regardless of the method used to calculate relative risk. Limiting the household vacuum cleaner samples to those with removable bags appears to increase the correlations between the two sampling methods, particularly for some of the PCB congeners.

## Conclusion

The household vacuum cleaner method appears to be a reasonable alternative to the HVS3 for detecting, ranking, and quantifying the concentrations of pesticides and other compounds, as long as dust loading is not a critical factor.

## Competing interests

The author(s) declare that they have no competing interests.

## Authors' contributions

JSC conducted the statistical analyses and drafted the manuscript. RBG participated in study design and helped draft the manuscript. CM participated in the NCCLS study coordination, statistical analyses, and preparation of the manuscript. MGN conducted the laboratory analyses. EMB participated in the design and coordination of the Fresno County study. PR participated in the design and coordination of the study. PAB participated in the NCCLS study conception and design, statistical analyses and preparation of the manuscript. MHW participated in the study design, statistical analyses, and preparation of the manuscript, and oversaw the laboratory analyses. All of the authors have read and approved the final version of the manuscript.
